# Skeletal Muscle Resident Progenitor Cells Coexpress Mesenchymal and Myogenic Markers and Are Not Affected by Chronic Heart Failure-Induced Dysregulations

**DOI:** 10.1155/2019/5690345

**Published:** 2019-01-03

**Authors:** R. I. Dmitrieva, T. A. Lelyavina, M. Y. Komarova, V. L. Galenko, O. A. Ivanova, P. A. Tikanova, N. V. Khromova, A. S. Golovkin, M. A. Bortsova, A. Sergushichev, M. Yu. Sitnikova, A. A. Kostareva

**Affiliations:** ^1^Institute of Molecular Biology and Genetics, National Almazov Medical Research Centre, Saint Petersburg, Russia; ^2^Heart Failure Department, National Almazov Medical Research Centre, Saint Petersburg, Russia; ^3^Peter the Great St. Petersburg Polytechnic University, Saint Petersburg, Russia; ^4^ITMO University, Saint Petersburg, Russia; ^5^Saint Petersburg State University, Saint Petersburg, Russia

## Abstract

**Background and Purpose:**

In heart failure (HF), metabolic alterations induce skeletal muscle wasting and decrease of exercise capacity and quality of life. The activation of skeletal muscle regeneration potential is a prospective strategy to reduce muscle wasting; therefore, the aim of this project was to determine if functional properties of skeletal muscle mesenchymal progenitor cells (SM-MPC) were affected by HF-induced functional and metabolic dysregulations.

**Methods:**

Gastrocnemius muscle biopsy samples were obtained from 3 healthy donors (HD) and 12 HF patients to purify mRNA for further analysis and to isolate SM-MPC. Cells were expanded in vitro and characterized by immunocytochemistry and flow cytometry for expression of mesenchymal (CD105/CD73/CD166/CD146/CD140b/CD140a/VIM) and myogenic (Myf5/CD56/MyoG) markers. Cells were induced to differentiate and were then analyzed by immunostaining and Q-PCR to verify the efficiency of differentiation. The expression of genes that control muscle metabolism and development was compared for HD/HF patients in both muscle biopsy and in vitro-differentiated myotubes.

**Results:**

The upregulation of MYH3/MYH8/Myf6 detected in HF skeletal muscle along with metabolic alterations indicates chronic pathological activation of the muscle developmental program. SM-MPC isolated from HD and HF patients represented a mixed population that coexpresses both mesenchymal and myogenic markers and differs from AD-MMSC, BM-MMSC, and IMF-MSC. The functional properties of SM-MPC did not differ between HD and HF patients.

**Conclusion:**

In the present work, we demonstrate that the metabolic and functional alterations we detected in skeletal muscle from HF patients do not dramatically affect the functional properties of purified and expanded in vitro SM-MPC. We speculate that skeletal muscle progenitor cells are protected by their niche and under beneficial circumstances could contribute to muscle restoration and prevention and treatment of muscle wasting. The potential new therapeutic strategies of HF-induced skeletal muscle wasting should be targeted on both activation of SM-MPC regeneration potential and improvement of skeletal muscle metabolic status to provide a favorable environment for SM-MPC-driven muscle restoration.

## 1. Introduction

In heart failure (HF), functional and metabolic alterations are detected not only in cardiac muscle [1, 2] but also in skeletal muscle tissue. Oxidative stress, systemic inflammation, chronic hypoxia, and decreased fatty acid oxidation coupled with mitochondrial dysfunction are the factors contributing to HF-induced muscle damage that include a shift in fiber type, induction of atrophy, development of insulin resistance, dysregulation of lipid metabolism, and ectopic fat depositions in the skeletal muscles. Additionally, chronic activation of adrenergic and natriuretic peptide systems in HF results in sustained lipolysis in adipocytes resulting in the accumulation of toxic and neutral lipid species in adipose and skeletal muscle that also contributes to skeletal muscle damage [3–9]. Impairments in skeletal muscle stem cell function have also been suggested as an important factor causing the loss of muscle mass with increasing age [10] and could similarly be considered as a factor contributing to HF-induced skeletal muscle wasting.

The development of preventive and therapeutic strategies against muscle wasting disorders remains an unresolved challenge. By now, exercise training, either alone or in combination with nutritional support, is the most proven strategy to reduce skeletal muscle wasting in HF patients and is recommended by treatment guidelines [7, 11]. Consequently, the activation of skeletal muscle developmental, growth, and regeneration potential is an essential mechanism to treat/prevent skeletal muscle wasting. Thus, the skeletal muscle progenitor cells that contribute to skeletal muscle regeneration and growth might be a prospective therapeutic target, and the analysis of the functional properties of skeletal muscle stem cells derived from heart failure patients has become a crucial issue.

Identification and characterization of myogenic progenitors in postnatal tissues are important for the evaluation of regeneration potential. In our recent work [12], we have demonstrated that bone marrow multipotent mesenchymal stromal cells (BM-MMSC) derived from heart failure patients are affected by heart failure in multiple ways: (1) in HF-derived cultures, we detected the upregulation of genes that control regeneration and fibrosis, including the Tgf-*β* pathway, synthesis of ECM, remodeling enzymes, and adhesion molecules; (2) during in vitro expansion, BM-MMSC from HF patients demonstrated early development of replicative senescence and decrease of proliferative activity; and (3) altered differentiation potential was also observed in HF-derived samples. However, when culturing conditions were modified, we have achieved the predominant purification and expansion of the highly proliferative nonprofibrotic CD146+/SMA*α* fraction that proves the potential efficacy of HF-derived BM-MMSC in regeneration processes [12].

Multipotent mesenchymal stromal cells are tissue-committed progenitors that preferentially contribute to the regeneration of certain types of tissue. For skeletal muscle, the role of nonsatellite resident myogenic progenitors including multipotent mesenchymal stromal cells in tissue regeneration was also reported [13–17]. In the current work, we sought to investigate whether or not HF-induced metabolic dysregulations affect the functional properties of resident skeletal muscle mesenchymal progenitor cells (SM-MPC) in order to determine if these cells could respond sufficiently to therapeutic interventions aimed at activating muscle regeneration and growth, including physical rehabilitation programs focused on the stabilization of muscle metabolism and prevention of skeletal muscle wasting.

## 2. Methods

### 2.1. Study Design and Ethical Issues

The current work is part of a complex ongoing project focused at evaluating the efficiency of aerobic physical training and developing personalized physical rehabilitation programs for heart failure patients. The first results that demonstrate the physiological response to aerobic physical training were published recently [18, 19]. During this project, the skeletal muscle biopsies should be taken from a selected group of patients enrolled in the program before and after a course of exercise training in order to examine the regenerative potential of muscle progenitor cells in HF patients as presented in this work; RNA-Seq analysis will be employed to reveal the global response of skeletal muscle to exercise training and determine potential specific targets when sample collection will be completed. In the current work, the first portion of the biopsy samples was used. All samples were collected under the agreement of the Institutional Ethics Committee at the Almazov National Medical Research Centre. All patients and donors entering the program agreed to and signed an institutional review board-approved statement of informed consent. The study was conducted in compliance with current Good Clinical Practice standards and in accordance with the principles under the Declaration of Helsinki (1989).

### 2.2. Human Subjects and Gastrocnemius Muscle Biopsy Samples

Only male donors were recruited into this study. A total of 3 healthy adult donors (HD) and 12 chronic heart failure patients (HF) were enrolled. HF patients have the following characteristics: NYHA II-III functional class, age 54 ± 12.5 years, body mass index (BMI) 26.5 ± 6.4 kg/m^2^, and left ventricle ejection fraction (LV EF) 26.4 ± 1.4%. The NYHA II : III patient ratio is 67% : 33%.

Inclusion criteria for the study are as follows: age 18–65 years, LV EF < 40% (Simpson), stable CHF NYHA II-III functional class, informed consent signing, ability to perform CPET, and optimal drug and electrophysiological therapy (ICD, CRT-D).

Exclusion criteria for the study are as follows: myocardial infarction and myocardial revascularization less than 3 months, stroke and CRT-D implantation less than 6 months, expressed cognitive impairment, any chronic disease decompensation, and high-gradation ventricular arrhythmias with no implanted cardioverter-defibrillator (ICD).

Gastrocnemius muscle biopsy samples were collected from each donor/patient at baseline and after 3–6 months of follow-up. Biopsy samples were divided into two portions. One was immediately transferred to liquid nitrogen for further mRNA purification. The second portion was used immediately for skeletal muscle progenitor cell purification.

Since our study was limited by the small number of HD enrolled into the project, we demonstrate the consistent response of HD-derived cells to stimulation of myogenic differentiation and similarities in immunophenotypes in supplemental Figure S1.

### 2.3. Purification and Separation of Skeletal Muscle Mesenchymal Progenitor Cells (SM-MPC) and Mesenchymal Stromal Cells from Intermuscular Fat (IMF-MSC)

SM-MPC were isolated enzymatically according to the protocols described previously [20, 21] with minor changes. In brief, isolated muscles were placed into an enzyme solution, mechanically disrupted with scissors, and digested for 60 min at 37°C in 5 mL filtered 0.1% collagenase I (C0130, Sigma-Aldrich, Germany). To remove collagenase and cell debris after digestion, the cell suspension was centrifuged for 5 min at 1000 × g and the supernatant was discarded. To release the stem cells from the fibers, the pellet was resuspended using sterile pipette tips in 2.5 mL of washing media (DMEM supplemented with 10% horse serum (HS) (Gibco, USA)). After the resuspension, the fibers were allowed to settle for 5 min and then the supernatant containing stem cells was transferred to a fresh tube. To increase the yield, this step was repeated twice. The double-collected supernatant was filtered through a 40 *μ*m nylon cell strainer and centrifuged for 10 min at 1000 × g in order to discard debris. Then, the resultant supernatant was discarded and the pellet of cells was placed in a proliferation media (DMEM supplemented with 10% FCS) on cell culture dishes and cultured until 80% confluence.

IMF-MSC samples were obtained from intermuscular adipose tissue located in biopsy material. IMF-MSC cultures were prepared as described in [22]. The separated sample of adipose tissue was washed with phosphate-buffered saline (PBS) and suspended in an equal volume of DMEM supplemented with 0.1% collagenase type III, prewarmed to 37°C. The tissue was placed in a shaking water bath at 37°C with continuous agitation for 30 min and centrifuged for 5 min at 300 × g at room temperature; then, the tissue sample was resuspended in culture media (DMEM supplemented with 10% FBC) and plated in a culture dish for expansion.

Bone marrow mesenchymal multipotent stromal cells (BM-MMSC) and subcutaneous adipose mesenchymal multipotent stromal cells (AD-MMSC) were collected, characterized, and saved as in our previous projects [22]. For this project, they were obtained from the biobank of the Almazov National Medical Research Centre, cultured as IMF-MSC, and used as control samples where appropriate.

### 2.4. Differentiation Protocols

Fusion of some cells without external stimuli usually was observed in subconfluent SM-MPC cultures and served as a reliable indicator, after which we induced skeletal muscle differentiation. To induce differentiation, the proliferation media was removed and replaced with differentiation media that was renewed after every other day. The DMEM media was supplemented with 2% of horse serum. Cultures were taken for experiments at day five and day seven after induction when myotubes were clearly visualized. Adipose tissue differentiation was stimulated as described earlier [22] by replacing the culture media with adipocyte induction medium composed of culture medium supplemented with 1 *μ*M insulin, 1 *μ*M dexamethasone, and 0.5 *μ*M 3-isobutyl-1-methylxanthine. Differentiated adipocytes were fixed and stained with Oil Red O at day 9 after induction.

### 2.5. Immunocytochemistry

The nature of the isolated cells was confirmed by immunocytochemical staining. Cells seeded onto cover glasses were fixed in 4% paraformaldehyde for 10 min at 4°C and then permeabilized with 0.02% Triton X-100 for 5 min. Nonspecific binding was blocked by incubation in 15% FCS for 30 min, followed by one-hour incubation with the following primary antibodies: anti-MyoG (R&D Systems, USA), anti-MYF5 (R&D Systems, USA), anti-vimentin (Sigma-Aldrich, USA), anti-CD146 (Sigma-Aldrich, USA), anti-desmin (D33, Dako, Denmark), myosin heavy chain (MF20, MAB4470, R&D Systems, USA), anti-myosin (skeletal fast; human MYH1/MYH2) (M4276, Sigma-Aldrich, USA), anti-myosin (skeletal slow; human MYH7) (M8421, Sigma-Aldrich, USA), and anti-myogenin (MAB6686, R&D Systems, USA). The secondary antibodies conjugated with Alexa Fluor 546/Alexa-488 (Molecular Probes, USA) were applied for 45 min at room temperature. Nuclei were counterstained with DAPI (Molecular Probes, USA).

### 2.6. Flow Cytometry Analysis

The immunophenotype of stem cells was evaluated by flow cytometry analysis performed on CytoFLEX (Beckman Coulter). Сells were resuspended in 100 *μ*L of PBS containing 1% of bovine serum albumin (Sigma-Aldrich, Saint Louis, MO, USA) and incubated for 20 min at 20°C in the dark with the following monoclonal antibodies (Ab): anti-CD56 PC7 (Beckman Coulter, USA, A21692), anti-CD146 PE (Beckman Coulter, USA, A07483), anti-CD166 PE (Beckman Coulter, USA, A22361), anti-CD73 PE (BD Pharmingen, USA, 550257), anti-CD105 APC (R&D Systems, USA, FAB1097A-100), anti-CD45 PC5 (Beckman Coulter, USA, A07785), anti-PDGFR*β* APC (BD Pharmingen, USA, FAB1263A), and anti-CD140a PE (BioLegend, USA, 323506). Data were analyzed using the CytExpert 2.0 (Beckman Coulter).

### 2.7. Cell Sorting

All sorting procedures were performed on a BD FACSAria™ III (Becton Dickinson, USA) flow cytometer using BD FACSDiva (Becton Dickinson, USA) software. Flow calibration was performed using Acudrop Beads (BD FACS™) with following stabilization for at least 25 minutes. The nozzle size was 100 mm, and the sheath pressure was set at 17 psi. During sorting, the flow rate was restricted to <800 events/sec to ensure minimal contamination. Additionally, a “4-way purity” sort option was used and is sufficient to gain a 99% pure sample. Before sorting commenced, appropriate settings were determined for all parameters.

Cell cultures were stained with antiCD56 PE (Beckman Coulter, USA, A07788) monoclonal antibodies according to the manufacturer's protocols. Primarily cells were detected in logarithmical scales in forward scattering (FS) and side scattering (SC). Sorting was performed according to the electronic gating strategy. Two target cell populations (CD56+ and CD56-, respectively) were collected in 15 mL falcon tubes containing PBS supplemented with 2% of FBS.

Sorting efficiency was controlled using additional flow cytometry analysis of sorted samples. Detected purity was no less than 95%.

### 2.8. RNA Isolation, cDNA Synthesis, and Q-PCR

Sequences for Q-PCR primers can be found in supplemental Table S1. Total RNA was isolated using the ExtractRNA reagent (Evrogen, cat. no. BC032, Russia). cDNA was synthesized from 500 ng of total RNA using a Moloney Murine Leukemia Virus Reverse Transcriptase MMLV RT kit (Evrogen, SK021, Russia). A quantitative evaluation of gene expression was performed with qPCR mix-HS SYBR+ROX (Evrogen, cat. no. PK156, Russia). Q-PCR data are presented as arbitrary units of mRNA expression normalized to *GAPDH* expression and to expression levels in the reference sample.

### 2.9. Statistical Methods

Statistical analysis was performed using GraphPad Prism 7 software. All data were analyzed with at least three biological replicates and presented as mean ± SEM. See figure legends for details for each specific experiment.

## 3. Results

### 3.1. Pathological Upregulation of Genes That Regulate Developmental/Regeneration Program and Metabolism Detected in Skeletal Muscle from HF Patients

**Figure 1:**
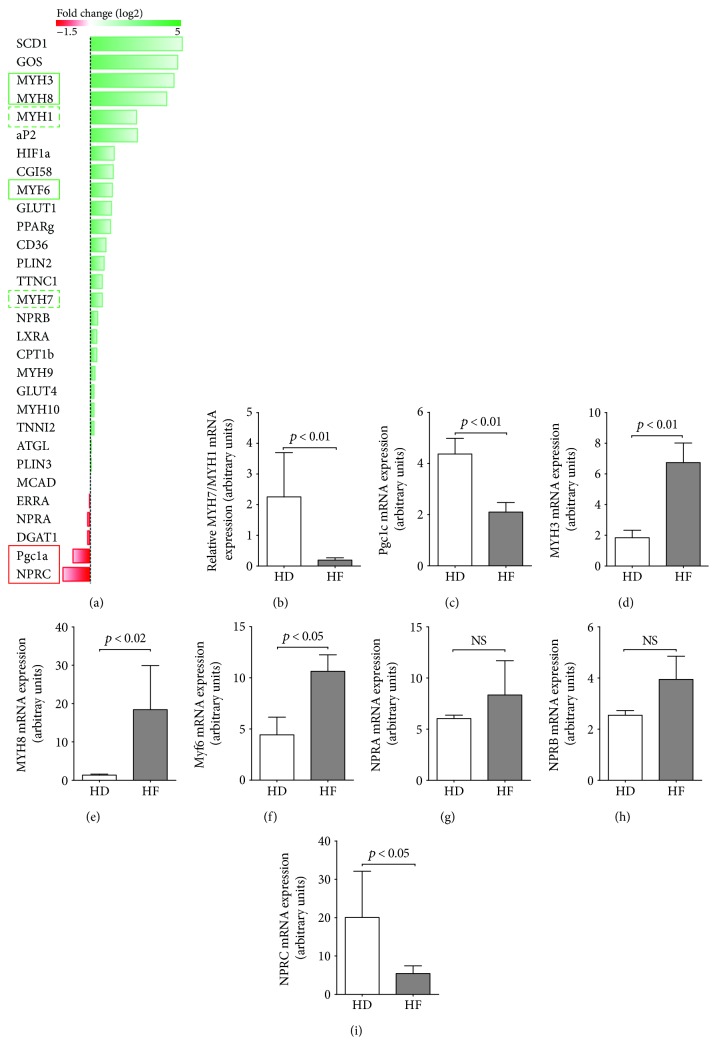
The expression of genes that regulate skeletal muscle development and metabolism is altered in HF patients. (a) Results of Q-PCR screening of key regulators and markers of skeletal muscle development and metabolism. Green bars: upregulation in HF; red bars: downregulation in HF; *n* = 3 (HD) and *n* = 12 (HF). (b–i) Results of Q-PCR analysis of mRNA expression for genes that demonstrated significant differences in between HD and HF. *n* = 3 (HD) and *n* = 12 (HF).

In order to detect markers of HF-induced functional and metabolic alterations in skeletal muscles, the expression analysis was performed in HD- and HF-derived biopsy samples for markers and regulators of skeletal muscle development, maturation and function (Myf6, Myh3,Myh8, Myh1, Myh4, Myh9, Myh10, Myh7, TNNI2, and TTNC1), and energy metabolism, including the expression of genes that regulate lipids and glucose handling (Pgc1a, HIF1a, GLUT1, GLUT4, aP2, PLIN2, PLIN3, PPARg, ATGL, SCD1, GOS, CGI58, CD36, NPRA, NPRB, and NPRC). A few genes from this panel demonstrated significantly altered expression in HF-derived samples (Figure 1(a)).

We have found that the balance between expression levels of slow oxidative skeletal muscle fiber MHC isoform MYH7 and fast glycolytic isoform MYH1 was notably downregulated in HF muscle (Figure 1(b)), along with the downregulation of the expression of Pgc1a (Figure 1(c)) that indicates an impairment in the regulation of the transcriptional program for mitochondrial biogenesis and oxidative metabolism in HF [23]. Furthermore, the expression levels of both developmental myosins, embryonic (MYH3) and neonatal (MYH8), were substantially upregulated in HF-derived skeletal muscle biopsies, which along with the upregulation of myogenic regulatory factor Myf6 detected in HF-derived samples (Figures 1(d)–1(f)) may indicate the chronic shift to developmental program and pathological stimulation of muscle regeneration in HF [24, 25].

We have also detected in HF skeletal muscle the alterations in the expression of the NPRA/B-to-NPRC ratio (Figures 1(g)–1(i)) that controls the biological activity of the natriuretic peptide system (NP) at the target tissue level [26], including control of sensitivity to insulin and lipid oxidative capacity through a Pgc1a-dependent pathway [27] and cardiac progenitor cell proliferation and differentiation into cardiomyocytes [28].

### 3.2. The Characterization of In Vitro Expanded SM-MPC Cells and Comparison with Mesenchymal Multipotent Cells from Different Sources

**Figure 2:**
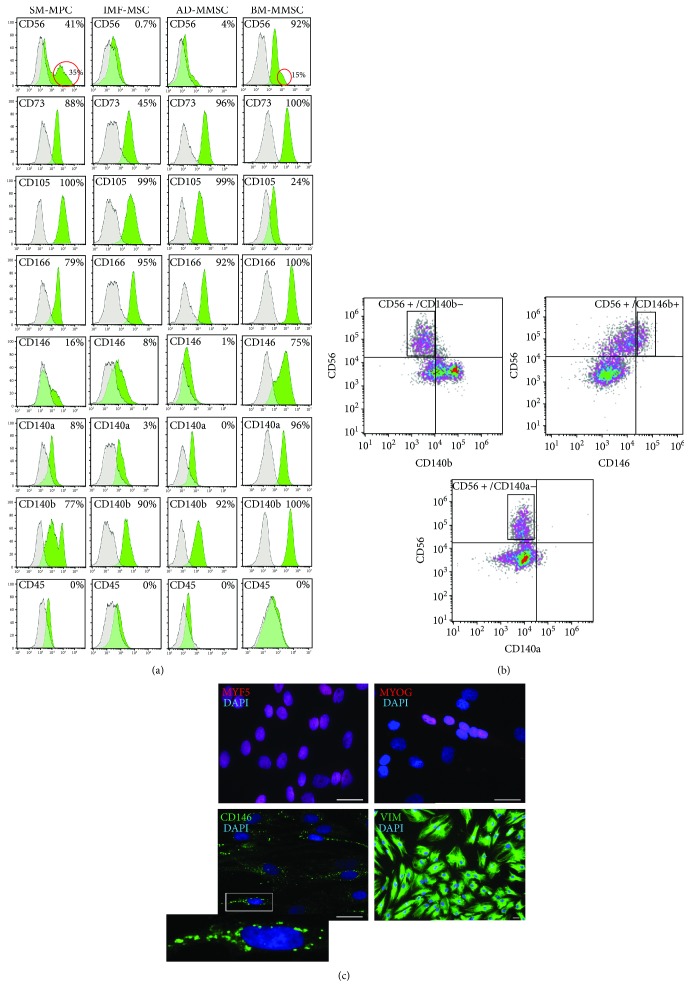
Characterization of SM-MPC, IFM-MSC, BM-MMSC, and AD-MMSC cultures expanded in vitro. (a) Representative histograms demonstrate the results of a comparative FACS surface marker analysis of SM-MPC, IFM-MSC, BM-MMSC, and AD-MMSC. Green histograms indicate the stained samples, and grey ones indicate the negative controls. The red ring indicates the CD56^bright^ subpopulations in SM-MPC and BM-MMSC samples (*n* = 3–5 in each group). (b) Visualization of FACS analysis in SM-MPC samples: CD56+/CD140a-, CD56+/CD140b-, and CD56+/CD146+ subpopulations are indicated in a rectangle gate; quadrant gates specify the negative/positive populations. Unstained cells were used as a negative control for each sample. (c) Immunocytological phenotyping of skeletal muscle precursor cells at early steps of expansion in vitro. Cells were stained for the expression of MYF5 (red; ~100% of positive), MYOG (red; 25± 4% of positive), vimentin (VIM, green; ~100 of positive), or CD146 (green; 20± 4.5% of positive). The insert demonstrates the enlarged region with a CD146+ cell. Nuclei were labelled with DAPI (blue). Scale bars represent 50 *μ*m.

Purified and expanded in vitro populations of SM-MPC derived from HD and HF muscle biopsies demonstrated a mixed phenotype that may change dynamically during in vitro expansion. Cells did not differ significantly from sample to sample and between HF patients and HD in both flow cytometry analysis and immunocytochemistry analysis. BM-MMSC, IMF-MSC, and AD-MMSC were used to demonstrate differences and similarities between muscle-derived stem cells and mesenchymal multipotent stromal cells from other sources. The representative images are presented in Figure 2.

Most of the SM-MPC cells were CD73+/CD105+/CD166+/CD140b+, and about 30–40% of the cells in the sample expressed NCAM/CD56, known as a reliable molecular marker of satellite cells and myoblasts in human skeletal muscle, as well as myotubes, and muscle fibers during development and/or regeneration [29] (Figure 2(a)). Interestingly, we detected two distinct subpopulations of CD140b^dim/bright^ cells in some samples, but none of these populations was CD56+ (Figure 2(b)). Furthermore, practically all SM-MPC cells demonstrated no CD140a expression and only about 15% of them were CD146^dim^. Markedly, a substantial fraction of CD146^dim^ cells was CD56+ (Figure 2(b)).

BM-MMSC demonstrated, as expected, the CD73+/CD166+/CD140a+/CD140b+/CD146+ phenotype and, surprisingly, expressed NCAM/CD56. The expression of CD56 on bone marrow-derived MSC is not common, but was reported previously at both mRNA and protein levels and was donor-specific with the CD56+ fraction ranging from 24 to 88.5% [30]. In our experiments in all 3 samples, we detected quite a big fraction of CD56+ cells; however, the fraction of CD56^bright^ cells in these samples was less than 15%, while in SM-MPC samples this fraction was more than 30% as indicated on Figure 2(a). Interestingly, unlike SM-MPC, in BM-MMSC samples all CD56+ cells were CD140a+/CD140b+.

The immunophenotypes of both fat-derived samples, IMF-MSC and AD-MMSC, were very similar and differ substantially from the ones of the SM-MPC and BM-MMSC samples. Virtually all cells in the IMF-MSC and AD-MMSC samples were CD56 negative and expressed mesenchymal markers CD73, CD105, CD166, and CD140b (PDGFRb), but they demonstrated little or no CD140a (PDGFRa) and CD146 expression.

The immunostaining analysis also revealed the coexpression of mesenchymal markers and markers of myogenic cells in SM-MPC (Figure 2(c)). Virtually all cells were expanded in vitro; however, the cells were not stimulated to differentiate into cells expressing early myogenic regulatory factor Myf5 [25], and some cells, presumably those that will undergo spontaneous fusion, expressed myogenin (MYOG) that regulates the fusion of myocytes and the formation of myotubes [25]. Furthermore, in vitro expanded skeletal muscle-derived stem cells expressed vimentin (Figure 2(c)), known to be expressed not only in mesenchymal cells [31] but also in myoblasts and in myotubes during early stages of embryonic development [32, 33]. The promyogenic nature of cells that express the melanoma cell adhesion molecule (MCAM, or CD146) was demonstrated recently [16], and we also have detected the CD146+ population in our samples (Figure 2(c)). We did Myf5 and MyoG immunostaining simultaneously for the same cultures that were stained for vimentin, and these data in combination with FACS analysis provide evidence of the coexpression of mesenchymal lineage cell markers with myogenic markers.

### 3.3. Both CD56+ and CD56- Fractions of SM-MPC Demonstrate Myogenic Potential

**Figure 3:**
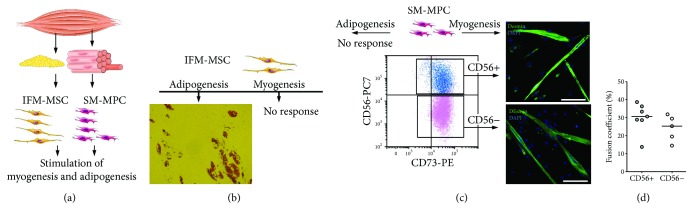
Comparative analysis of the functional properties of subpopulations of skeletal muscle progenitor cells derived from muscle tissue (SM-MPC) and from intermuscular fat (IMF-MSC). (a) The design of the experiment is as follows: SM-MPC and IMF-MSC were purified from muscle biopsy, expanded in vitro, characterized, and induced to differentiate. (b) IMF-MSC under appropriate stimulation undergo adipogenesis but do not respond to promyogenic stimulation. (c) Both CD56+ and CD56- fractions of SM-MPC demonstrate the ability to differentiate into myotubes but do not respond to adipogenic stimuli; scale bars represent 100 *μ*m. (d) Fusion coefficient is calculated as a percent of nuclei incorporated in MF20+ myotubes, and it does not differ between the CD56+ and CD56- subpopulations.

In order to determine if both major subpopulations of SM-MPC (CD56-/CD56+) possess the myogenic potential and may therefore be important for the maintenance of skeletal muscle regeneration, we employed FACS sorting to separate these subpopulations from SM-MPC; purified CD56-/CD56+ samples were expanded in vitro and induced to differentiate into adipose and muscle tissue (Figure 3). IMF-MSC samples were used as a control.

As we expected, IMF-MSC samples did not respond to the stimulation of myogenesis but were differentiated actively into adipocytes (Figure 3(b)). On the contrary, both subpopulations of SM-MPC did not differentiate into adipocytes under adipogenic stimuli but demonstrated the ability to differentiate into myotubes (Figure 3(c)). The fusion coefficient was slightly but not significantly higher in the CD56+ subpopulation (30 ± 3% vs 24 ± 35) (Figure 3(d)), which confirmed the potential significance of both fractions of SM-MPC for muscle regeneration/development. Taking into account all information mentioned above, we choose to use the whole unsorted HD- and HF-derived SM-MPC cellular samples in further work.

### 3.4. Immunohistochemical Analysis and Gene Expression Analysis during HD- and HF-Derived SM-MPC Differentiation In Vitro Did Not Reveal Differences between Groups

**Figure 4:**
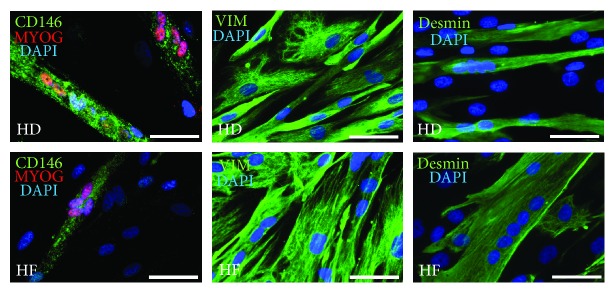
Immunocytological phenotyping of differentiated myotubes at early steps of myogenesis. At day 5 after stimulation, myotubes coexpress myogenin (MYOG) and CD146. All cells in culture express vimentin (VIM), and desmin expression is specifically associated with multinucleated myotubes. Scale bars represent 50 *μ*m.

HD- and HF-derived SM-MPC demonstrated a similar ability to respond to the stimulation of myogenesis in vitro. We have done immunocytochemical staining at early and late steps of differentiation. At day five after induction, the formation of myotubes was observed and immunocytochemical staining detected the expression of myogenin, CD146, and vimentin. It is known that vimentin is the most abundant intermediate filament protein in immature myoblasts/muscle progenitors. During the early steps of muscle development, desmin and vimentin are coexpressed. Upon further differentiation into mature muscle cells, desmin is strongly upregulated, while the expression of vimentin completely ceased [34]. In our cultures, we detected vimentin in progenitor cells (Figure 2(c)) and in myotubes at the early steps of differentiation (Figure 4), while desmin expression was detected in all tested conditions (Figures 3 and 4).

**Figure 5:**
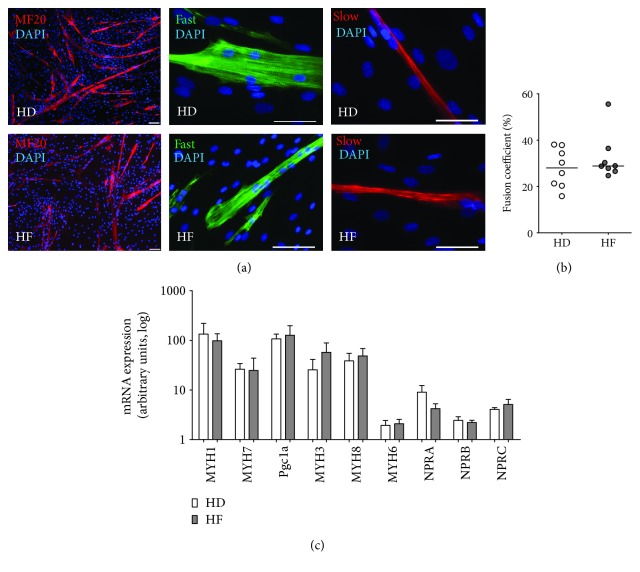
Differentiated myotubes do not differ significantly between HD- and HF-derived skeletal muscle progenitor cells. (a) At day 7 after stimulation, myotubes were stained for the expression of MyHC with an antibody that recognizes the heavy chain of myosin II (MF20) and markers of slow MYH7 and fast MYH1/MYH2 fibers. Nuclei were labelled with DAPI (blue). Representative images are given for both HF- and HD-derived samples. Scale bars represent 50 *μ*m. (b) Fusion coefficient is calculated as a percent of nuclei incorporated in MF20+ myotubes at day 7 after stimulation, and it does not differ between HD- and HF-derived samples. (c) mRNA expression analysis was performed for key markers of muscle development and metabolism for both HF- and HD-derived samples.

Staining for desmin (Figure 4) and MF20 (data not shown) at day 5 did not demonstrate a cross-striated pattern specific for mature skeletal muscle fiber (Figure 4). At day seven of immunocytochemical staining with antibodies against MF20, slow and fast MyHCs confirmed that differentiated myotubes demonstrate a cross-striated pattern, similar to that seen in adult muscle fibers. Both HD- and HF-derived cells developed myotubes that were positive for both slow (MYH7) and fast (MYH1/MYH2) myosins, and the fractions of nuclei incorporated into myosin-positive myotubes did not differ significantly between HF and HD samples (Figure 5(a)). The fusion coefficient did not differ significantly between HD and HF samples (28 ± 4% vs 32 ± 5%) (Figure 5(b)).

The gene expression analysis of markers and regulators of myogenic differentiation of SM-MPC derived from HD and HF patients confirmed the results of immunohistochemistry: the expression of slow skeletal muscle fiber MHC isoform MYH7 and fast isoform MYH1 did not differ significantly between HD- and HF-derived differentiated myotubes, and the expression of embryonic (MYH3) and neonatal (MYH8) myosins did not differ significantly as well. The expression of myogenic regulatory factor Myf6, Pgc1a, and of atrial natriuretic peptide receptor C in differentiated myotubes also did not differ significantly between groups (Figure 5(c)).

## 4. Discussion

Heart failure is a multiorgan syndrome affecting different cell types, including skeletal muscle. The development of preventive and therapeutic strategies against muscle wasting disorders in HF remains an unresolved challenge, and activation of developmental/regeneration programs in skeletal muscle could be considered as a prospective approach. Therefore, the activation of skeletal muscle progenitor cells would be beneficial for the restoration of skeletal muscle structure and performance. In this work, we aimed to determine if HF-induced skeletal muscle alterations affect SM-MPC developmental/regeneration potential.

We have detected a number of chronic dysregulations in the skeletal muscle tissue from our patients. The most important one was the chronic activation of the developmental program: the expression of mRNA of MYH3/MYH8 myosins and myogenic regulatory factor Myf6 was detected in an HF-derived biopsy. These alterations in combination with changes in slow/fast fiber composition may severely influence skeletal muscle metabolism, structure, and performance. We suggest that in HF, the regeneration process is stimulated in response to HF-induced damage: skeletal muscle progenitor cells progress successfully through activation, proliferation, differentiation into myoblasts, and fusion steps; however, instead of advancing to the fiber maturation stage, they get “stuck” at the developmental phase presumably due to chronic metabolic alterations observed in HF skeletal muscle. The reexpression of developmental myosins in adult skeletal muscle was detected in different pathological conditions that involve muscle degeneration/regeneration, such as trauma, chronic denervation, muscular dystrophy, and different types of myopathies (reviewed in [24]); however, to our knowledge none was previously reported for HF.

The shift from oxidative fiber type I to glycolytic fiber type II and reduced oxidative enzyme activities is the best-described HF-induced metabolic alteration in skeletal muscle [3, 5, 8, 35, 36]. We have also detected the shift in the expression ratio between fiber type I and fiber type II in skeletal muscle from HF patients, as well as the downregulation of expression of Pgc1a (Figure 1), which is an important mediator of mitochondrial metabolic properties in skeletal muscle and is downregulated in various types of atrophying muscle [37] including skeletal muscle of rats with HF [38–41]. Furthermore, HF is a state of chronic activation of adrenergic and NP systems, which besides their well-documented role in the cardiovascular system also plays a role in favoring fat oxidative capacity in human skeletal muscle cells [42] via the activation of cGMP signaling, induction of PGC1a, and enhancement of mitochondrial respiration [27]. There are also recent data showing that the NP system is involved in the regulation of cardiac progenitor cell proliferation via NPR-A and differentiation into cardiomyocytes via NPR-B [28] contributing to heart development and regeneration. The switch in expression balance from NPR-A/B to NPRC detected in our work indicates an increase of NP system activity [9] that could also impact on the upregulation of developmental signaling in HF skeletal muscle. Together, we have detected a number of alterations in HF-derived skeletal muscle that would affect developmental, metabolic, structural, and functional properties of skeletal muscle.

Next, we purified SM-MPC from HD- and HF-derived biopsies and investigated if the functional properties of HF-derived cells were affected by these alterations. The SM-MPC that we isolated from skeletal muscle biopsy samples from HD and HF patients represented a mixed population of cells that express both mesenchymal and myogenic markers. Samples were characterized by FACS analysis (Figures 2(a)–2(c)) and immunocytochemistry (Figure 2(c)), and they were also investigated for the ability to differentiate in vitro (Figure 3). In order to better evaluate the myogenic potential of muscle progenitor cells derived from HD and HF subjects based on obtained results, we concluded using the whole unsorted samples in differentiation experiments in order to retain in cultures all subpopulations that could possibly contribute to stimulated in vitro myogenesis either via paracrine signaling mechanisms and/or direct cell-cell interaction.

Indeed, there is a lot of evidence in the literature that describes different subpopulations of skeletal muscle progenitor cells that support skeletal muscle development, growth, and regeneration (reviewed in [13]). The best characterized myogenic progenitors in postnatal muscle are satellite cells that are activated in response to injury or stimulation to growth, which then start to proliferate and generate a pool of myoblasts able to fuse into newly formed myofibers. CD56 is considered as the most reliable satellite cell surface marker [38]. However, human satellite cells are not easy to isolate, purify, and expand in culture: most of the studies with satellite cells were done on mice, but not on humans [16]. Furthermore, in recent years reports of myogenic cells distinct from satellite cells have accumulated, and not all of these cells are reported to be CD56+. For example, PW1+/Pax7– interstitial cells (PICs) that do not express CD56 but demonstrate bipotential behavior in vitro, generating both smooth and skeletal muscles, were isolated and characterized [17]; the coexpression of mesenchymal (CD90, CD73, CD166, and CD105) and myogenic (CD56) markers was reported on several cell populations with myogenic potential including human embryonic mesodermal progenitors [43], expanded in vitro muscle-derived primary cultures [14], and myogenic mesenchymal progenitors derived from hES and iPSC [29]. It was also shown that subendothelial- (mural) cultured CD146+ cells (also known as “mesenchymal stem cells”) purified from various groups of skeletal muscle were able to spontaneously generate myotubes in vitro and myofibrils in vivo, and the expression of CD146 and CD56 was mutually exclusive in distinct myogenic cell subsets, with no coexpression [16]. Importantly, in this work the authors report that sorted and cultured CD146+ human muscle-derived cells progressively turn on the expression of myogenic markers PAX7, PAX3, Myf5, CD56, desmin, and MyHC, as they were verified by fluorescent immunocytochemistry [16]. Finally, in some protocols CD146 is recommended as a positive selection marker in cell sorting to obtain a human fetal myoblast population [44]. Together, all these previous findings support observations made in our work. Firstly, in expanded in vitro adherent cells purified from gastrocnemius muscle biopsy we also detected substantial subpopulations of cells that coexpress mesenchymal and myogenic markers (Figure 2). Secondly, we have found that not only the sorted CD56+ but also the sorted CD56- subpopulation demonstrates myogenic potential (Figure 3). Because of the limited volumes of samples, we were not able to monitor the dynamics of marker expression during in vitro expansion of sorted CD56+/CD56- populations; however, we can speculate that the myogenic potential of the CD56- subpopulation can be related to the CD146+ subpopulation of cells and those cells could turn on the expression of myogenic markers in the course of expansion as described by Persichini et al. [16]. The data presented on Figures 2 and 3 support this speculation pretty well: the coexpression of CD146 and CD56 on certain subpopulations of expanded in vitro SM-MPC (Figures 2(a) and 2(c)) and coexpression of myogenic regulatory factor myogenin (MyoG) with CD146 at early steps of myogenic differentiation (Figure 3) confirm the importance of the CD146+ SM-MPC fraction for myogenic differentiation.

Also, as indicated on Figure 3, both major fractions of SM-MPC (CD56+/CD56-) demonstrated a similar ability to differentiate into myotubes but not into adipocytes. This is an important observation: dysregulation of lipid metabolism in the skeletal muscles of HF patients is a well-described metabolic disorder [9], and fatty degeneration of skeletal muscle is often associated with metabolic dysregulation [45, 46]. In our experiments, neither HD nor HF-derived SM-MPC demonstrated in vitro adipogenic potential. Our data fit well the observations described by Uezumi et al. [45] who demonstrated that only the CD140a+ mesenchymal progenitor population of muscle-derived cells show efficient adipogenic differentiation both in vitro and in vivo. In our work, SM-MPC demonstrated a CD140a- phenotype. Interestingly, both adipose tissue-derived AD-MMSC and IMF-MSC, but not BM-MMSC samples that all differentiate into adipocytes (Figure 2, our previous data [22]), demonstrated a CD140a- phenotype.

Interestingly, we have detected myotube formation not only upon canonical differentiation conditions (2% horse serum), but even under adipogenic stimulation (data not shown). Similar observations were made previously by others: myogenesis in skeletal muscle-derived progenitor cells under adipogenic stimulation was mentioned earlier by Persichini et al. [16] and by Uezumi et al. [45]; those data, however, did not get much attention and deserve further investigation. Together, these observations allow us to conclude that SM-MSC samples purified from both HD- and HF-derived skeletal muscle were restricted/committed to myogenic differentiation and, in general, do not differ between healthy donors and heart failure patients.

## 5. Conclusion

In the present work, we demonstrate that the metabolic and functional alterations we detected in skeletal muscle from HF patients do not dramatically affect the functional properties of purified and expanded in vitro skeletal muscle mesenchymal progenitor cells (SM-MPC).

These findings allow us to speculate that skeletal muscle progenitor cells are quite well protected by their niche from HF-induced metabolic stress, and they could, under beneficial circumstances, contribute to damaged muscle restoration, prevention, and treatment of muscle wasting; the exact mechanisms behind alterations in the muscle regeneration program in HF remain to be investigated, and the potential new therapeutic strategies of HF-induced skeletal muscle wasting should be targeted on both activation of skeletal muscle stem cell regeneration potential and improvement of skeletal muscle metabolic status in order to provide a favorable environment for SM-MPC-driven improvement of muscle structure and performance.

## Data Availability

The data used to support the findings of this study are available from the corresponding author upon request.

## Supplementary Materials

Supplementary Materials Table S1: list of primers used in Q-PCR experiments. Figure S1: SM-MPC from HD demonstrate the consistent response to stimulation of myogenic differentiation and similarities in immunophenotypes. Upper panel: immunocytological visualization of differentiated myotubes in HD-derived samples: myotubes were stained for expression of MyHC with an antibody that recognizes the heavy chain of myosin II (MF20). Nuclei were labelled with DAPI (blue). Lower panel: histograms demonstrate results of FACS surface marker analysis of SM-MPC derived from HD.
