# Supplementary material for: Skeletal Muscle Resident Progenitor Cells Coexpress Mesenchymal and Myogenic Markers and Are Not Affected by Chronic Heart Failure-Induced Dysregulations
Source: Stem Cells Int. 2019 Jan 3;2019:5690345. doi: 10.1155/2019/5690345 (PMC6335669; doi:10.1155/2019/5690345)
Supplement: Supplementary Materials — Table S1: list of primers used in Q-PCR experiments. Figure S1: SM-MPC from HD demonstrate the consistent response to stimulation of myogenic differentiation and similarities in immunophenotypes. Upper panel: immunocytological visualization of differentiated myotubes in HD-derived samples: myotubes were stained for expression of MyHC with an antibody that recognizes the heavy chain of myosin II (MF20). Nuclei were labelled with DAPI (blue). Lower panel: histograms demonstrate results of FACS surface marker analysis of SM-MPC derived from HD. [file 5690345.f1.pdf]

Table S1. List of primers used in Q-PCR experiments

GENE NAME	Forward primer	Reverse pimer
hGAPDH	AATGAAGGGGTCATTGATGG	AAGGTGAAGGTCGGAGTCAA
hMYH1	CACCACCAACCCATACGATTA	GGCACTATCTGTAGCCATCAA
hMYH3	GTGGTGGACTCAAAGGAAGAA	GGTCCTGTTGTCCTCAGTTT
hMYH7	GACCTCAAGAAGGATGTCTTCG	GGTCACTGTCTTGCCATACTC
hMYH8	TTTCCACCAAGAACCCAGAG	CACTCATGGCTGCGATTTATTT
hMYH9	CAGGGCTCATCTACACCTATTC	GCCCTTGTACATTTCCACAATC
hMYH10	GGGCCAACATTGAAACATACC	TTCTCCTGCTCCAGATAACAAC
TNNC1	AGAGCAGCTGACAGAAGA	GCTCCTTGGTGCTGATG
hMYH6	CACCAACAATCCCTACGACTAC	AGCACGTCAAAGGCACTATC
TNNI2	CATGTCTGAAGTGCAGGAG	AGCTCCTTGCTGGTCTT
hPGC-1a	GCTCTGTGTCACTGTGGATT	CACACTCGATGTCACTCCATAC
hSCD1	AGGACGATATCTCTAGCTCCTATAC	GGCATCGTCTCCAACTTATCT
hGOS	CCGTGCCACTAAGGTCATT	ACGTACAGCTTCACCATCTTC
hCD36	GGCTGTGTTTGGAGGTATTCTA	TTGTACCTTCTTCGAGGACAAC
hPLIN2	CAGTAGTCGTCACAGCATCTT	CAGTAGTCGTCACAGCATCTT
hPLIN3	ACACAAGCTCCTTGGTGTC	ACACAAGCTCCTTGGTGTC
hLXRa	TTGGACAGTGCCTTGGTAAT	CGCAGAGTCAGGAGGAATG
hCPT1b	GCTATGTGTATCCGCCTTCTATC	GACTCTAGGTACCGCTGAATTG
hCGI58	CAGCGTTTAAGGCCTGATTTC	CCACTTGGAGTCTGCACATTA
hATGL	TGTCTGCAGCGGTTTCAT	CTCATAGAGTGGCAGGTTGTC
hMCAD	CAGATCCTAAAGCTCCTGCTAAT	GGCCCATGTTTAATTCCTTTCTC
hERRa	ATGGTGTGGCATCCTGTG	CTTGGTGATCTCACACTCGTT
hDGAT1	CTGGAGAACCTCATCAAGTATGG	CAAAGACATTGGCCGCAATAA
hPPARa	CAAGGCCTCAGGCTATCATTAC	GTCGCACTTGTCATACACCA
hPPARg	AGCCTTCCAACTCCCTCATGGCA	TCCGGAAGAAACCCTTGCATCCT
hGLUT1	GACGTAGGGACCACACAGTTGC	CCAGCTGCCATTGCCGTT
NPRA	CGTGTGAACCGTAAACGCATT	CATTCTGCACATCCCGCATA
NPRB	TTCAAGGGAAATGTTGTCGCC	AACAGAACCTGCCGGGTCA
NPRC	CCCAGGAGGTTATTGGTGATTATTT	ACATTCGGCCGCATTTCAA
aP2	GCCAGGAATTTGACGAAGTC	TGGTTGATTTTCCATCCCAT

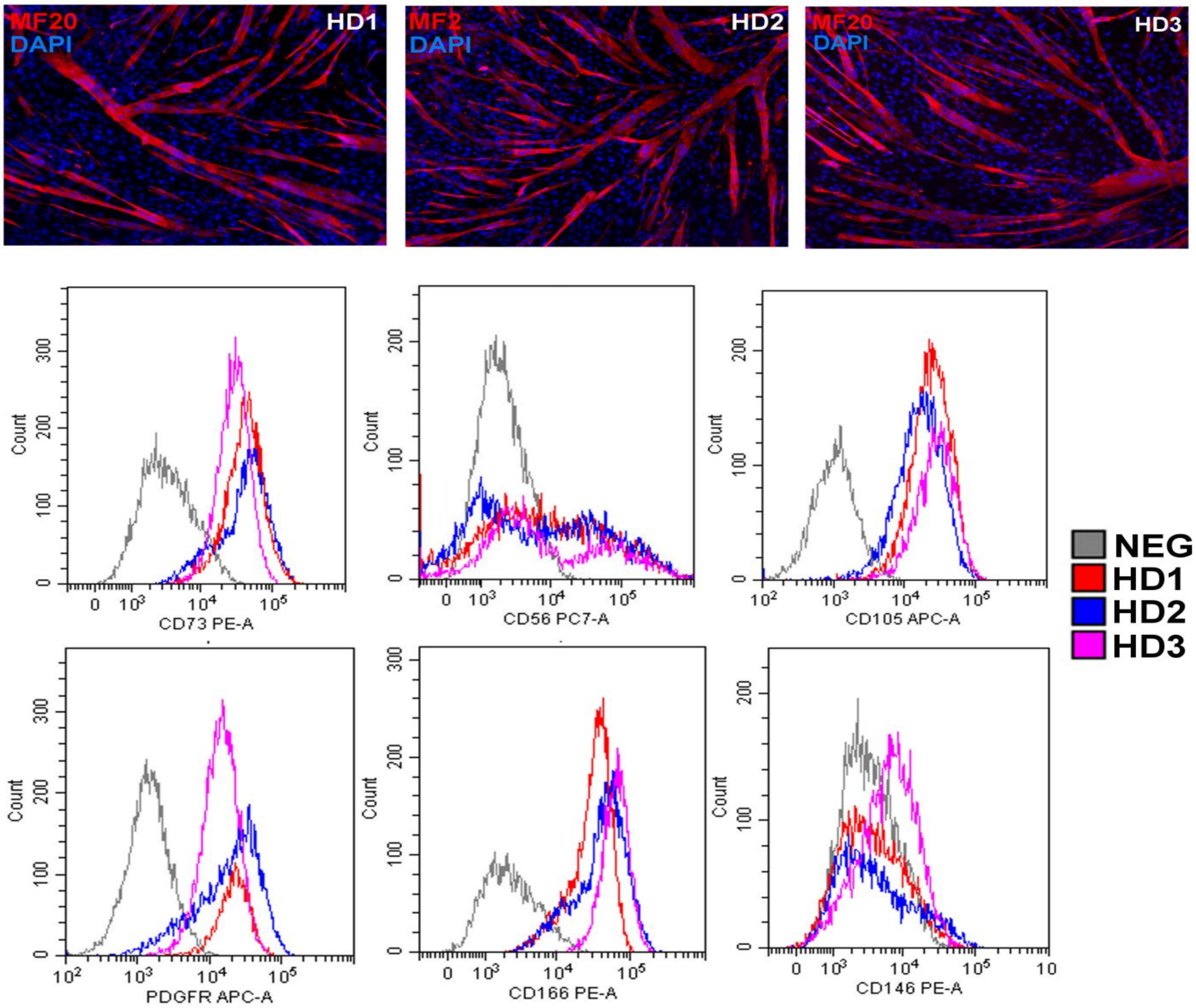

Figure S1. SM-MPC from HD demonstrate the consistent response to stimulation of myogenic differentiation and similarities in immunophenotypes.

Upper panel: Immunocytological visualization of differentiated myotubes in HD - derived samples: myotubes were stained for expression of MyHC with antibody that recognize the heavy chain of myosin II (MF20). Nuclei were labelled with DAPI (blue).

Lower panel: Histograms demonstrate results of FACS surface marker analysis of SM-MPC derived from HD.
